# High‐risk pulmonary embolism with out‐of‐hospital cardiac arrest: Acute multidisciplinary approach leading to surgical embolectomy with good clinical outcome

**DOI:** 10.1002/ccr3.3212

**Published:** 2020-08-28

**Authors:** Morten Engholm, Asger Andersen, Hans Eiskjær, Kaj Erik Klaaborg, Troels Thim

**Affiliations:** ^1^ Department of Cardiology Aarhus University Hospital Aarhus Denmark; ^2^ Department of Cardiothoracic Surgery Aarhus University Hospital Aarhus Denmark

**Keywords:** acute pulmonary embolism, cardiac arrest, cardiogenic shock, case report, multidisciplinary team conference, pulmonary angiography, pulmonary embolectomy

## Abstract

Percutaneous pulmonary angiography may be used for early diagnosis of pulmonary embolism in the hemodynamic unstable patient. Pulmonary embolectomy is an effective treatment option in patients with acute high‐risk pulmonary embolism.

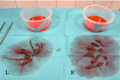

## 1 INTRODUCTION

A 72‐year‐old male presented with out‐of‐hospital cardiac arrest. Return of spontaneous circulation was obtained following cardiopulmonary resuscitation. He remained unstable and was admitted to the catheterization laboratory. Echocardiography showed acute cor pulmonale, and pulmonary angiography revealed massive pulmonary embolism. Surgical pulmonary embolectomy was performed with a good clinical outcome.

## 2 HISTORY OF PRESENTATION

**Figure 1:**
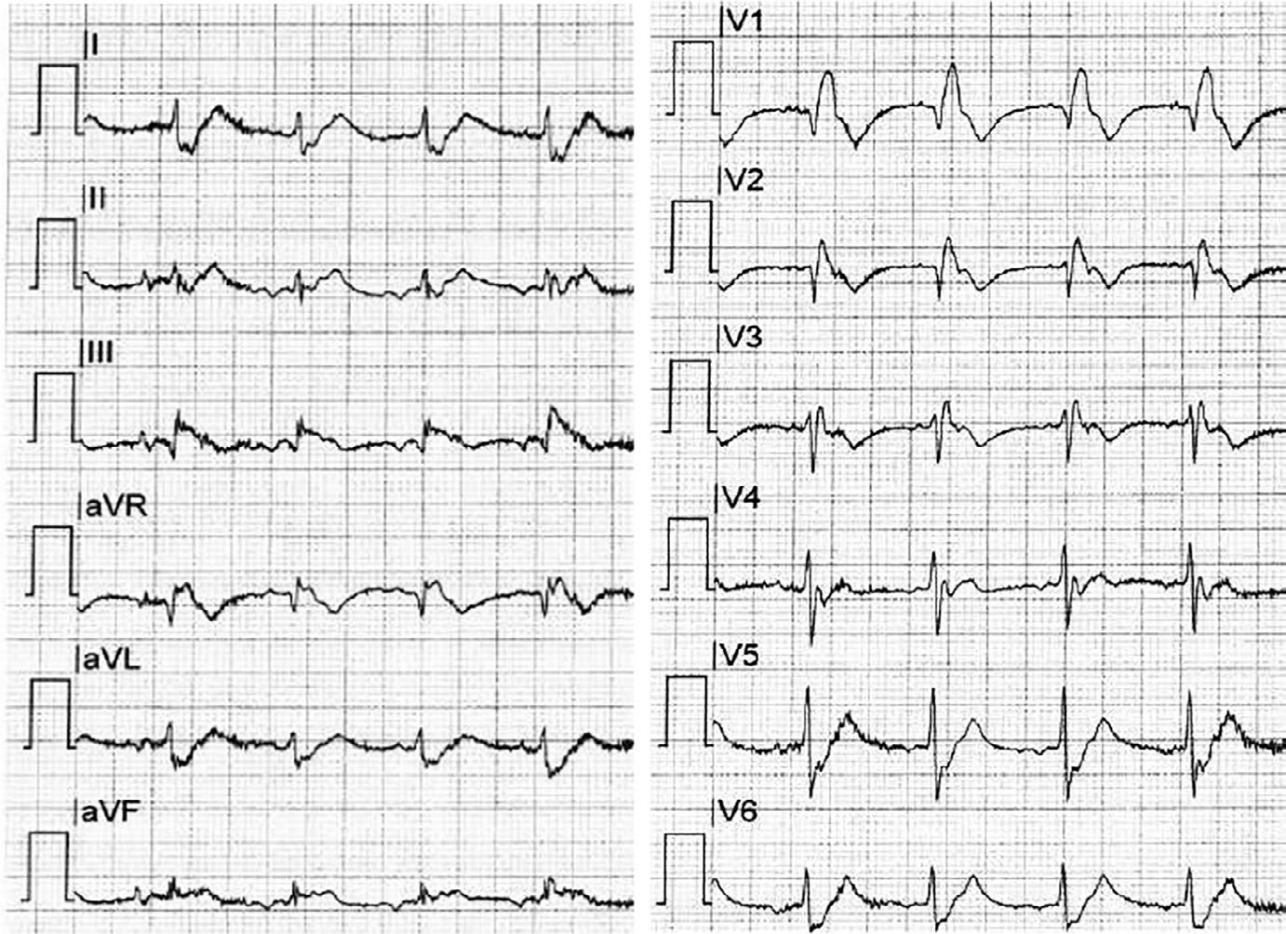
Pre‐hospital electrocardiogram

A 72‐year‐old male, current smoker with no previous history of cardiac disease, presented with acute onset of breathlessness and chest pain. Following emergency call and upon arrival of the ambulance response team, his clinical condition acutely deteriorated to out‐of‐hospital cardiac arrest (OHCA) with pulseless electrical activity (PEA). Prehospital cardiopulmonary resuscitation (CPR) was provided according to European Resuscitation Council (ERC) 2015 Guidelines for advanced life support. Intravenously adrenaline (1 mg) was administered during CPR and patent airway secured by endotracheal intubation and establishment of mechanical ventilation. Return of spontaneous circulation (ROSC) was obtained prehospitally after 3 minutes of CPR. Prehospital ECG showed sinus tachycardia 120 BPM with right bundle branch block (RBBB) and S1Q3T3 pattern (Figure 1).

Because of unstable hemodynamics and possible need of advanced mechanical support, the patient was admitted directly to the catheterization laboratory, following teleconference between the prehospital medical services and the tertiary heart center. He was transported 35 miles by the emergency medical services (Table 1).

**Table 1:** Timeline

Time	Events
12:15	Acute onset of dyspnea and chest pain.
12.30	Emergency medical service alarm call.
12:50	Arrival of ambulance. Paramedics find a critically ill, wet and cold patient with dyspnea and cyanosis. Blood pressure (BP) 75/49 mm Hg. Heart rate (HR) 121 BPM. Respiratory rate 37 breaths per minute. SpO_2_ 88%.
12:55	Cardiac arrest. Primary rhythm was pulseless electric activity (PEA). Cardiopulmonary resuscitation.
12:58	Return of spontaneous circulation (ROSC).
14:48	Arrival at the cardiac catheterization laboratory. BP 90/60 on vasopressor support with epinephrine. Heart rate 120 BPM. SpO_2_ 99%. ETCO2 2.5. Lactate level of 8.8 mmol/L and pH 7.12.
14.52	Cardiac arrest with PEA. ROSC following brief CPR and adrenaline. Echocardiography with acute cor pulmonale. Coronary angiography without culprit. Pulmonary angiography showed massive pulmonary embolism.
15.20	Multidisciplinary team conference in the catheterization laboratory.
15.25	Transfer to cardiothoracic operating room for surgical pulmonary embolectomy.
18.00	Transfer to ICU for postoperative stabilization.

Upon admission to the catheterization laboratory, the patient was intubated and sedated with Propofol plus Fentanyl. He was in cardiogenic shock with noninvasive blood pressure of 90/60 mm Hg on vasopressor support with epinephrine. Clinical examination showed tachypnoea and perioral cyanosis. There were accelerated regular heart sounds without murmurs and normal pulmonary auscultation. Arterial blood gas showed elevated lactate level of 8.8 mmol/L and pH 7.12. His clinical condition deteriorated rapidly on the catheterization laboratory table with progressive hemodynamic instability, hemodynamic collapse, and cardiac arrest. ROSC was achieved following a brief period of CPR and 1 mg intravenous adrenaline. Cumulative time of CPR (out‐of‐hospital and in‐hospital) was 10 minutes. Supportive vasopressor and inotropic therapy was established. Backup extracorporeal membrane oxygenation (ECMO) was simultaneously prepared, but never initiated.

## 3 INVESTIGATIONS

**Figure 2:**
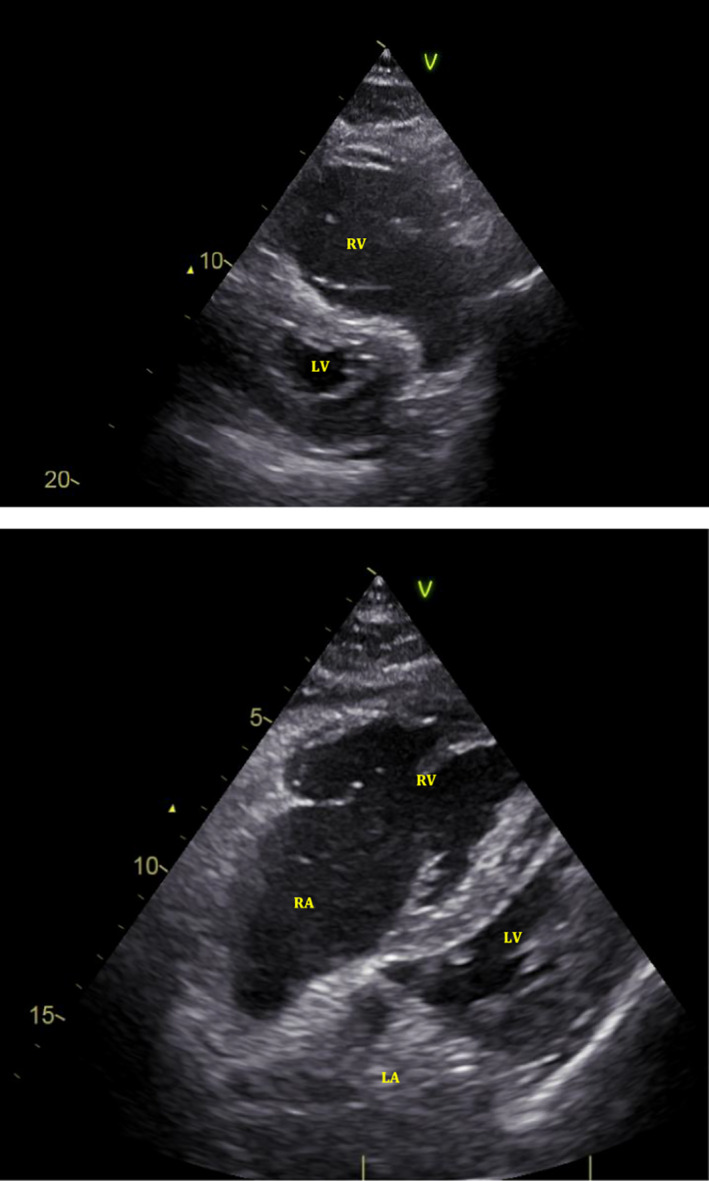
Echocardiography demonstrated massive dilatation of right heart chambers and acute right ventricular failure, with positive McConnell's sign. LA; left atrium, LV; left ventricle, RA; right atrium, and RV; right ventricle

**Figure 3:**
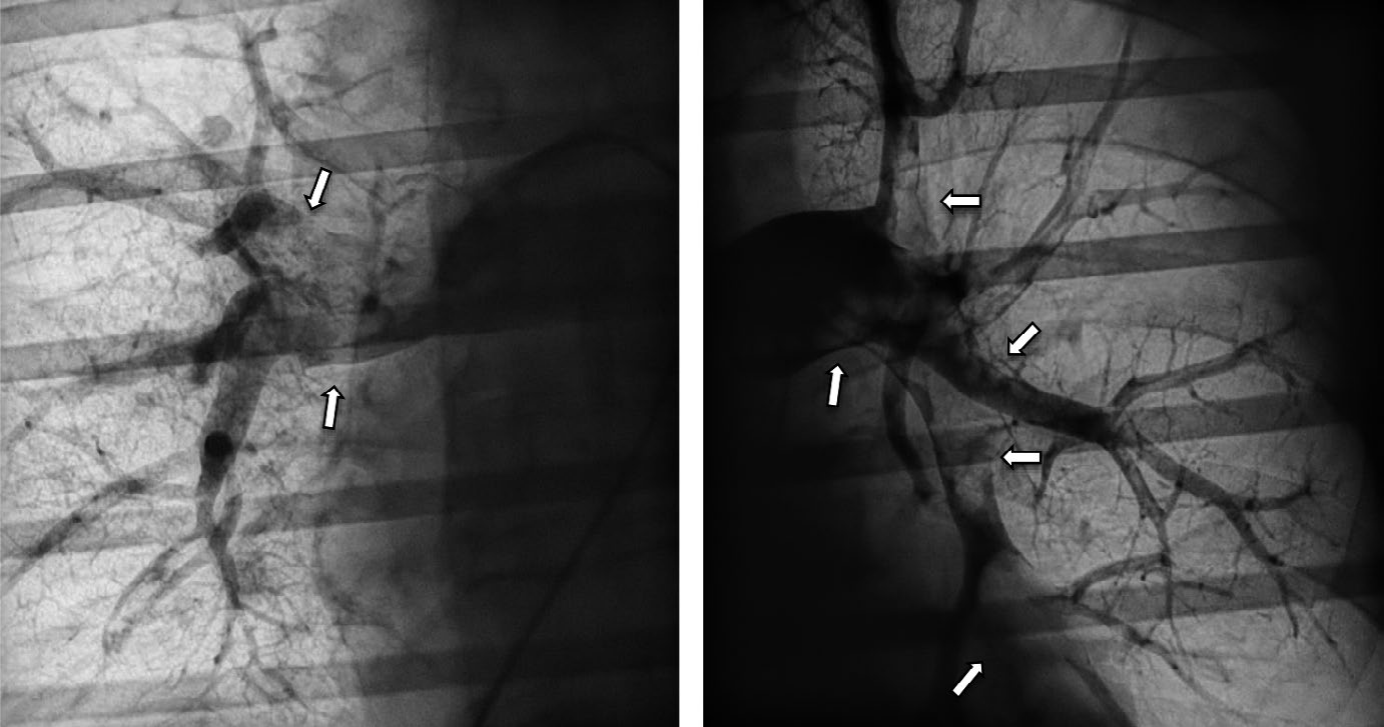
Pulmonary artery angiography confirmed severe intraluminal filling defects and missing pulmonary arterial branches caused by central pulmonary embolism (white arrows)

Echocardiography demonstrated massive right ventricular (RV) dilation and acute RV failure (Figure 2), suggesting low cardiac output secondary to massive pulmonary embolism (PE). Coronary angiography showed moderate diffuse coronary artery disease, but no culprit lesion. Additional pulmonary artery angiography confirmed severe intraluminal filling defects with central obstruction of the pulmonary arteries by thrombus material (Figure 3).

## 4 MANAGEMENT

The cardiac surgeons were called for acute multidisciplinary team (MDT) conference in the catheterization laboratory. It was decided to perform surgical pulmonary embolectomy, and the patient was then transferred directly to the operating room.

**Figure 4:**
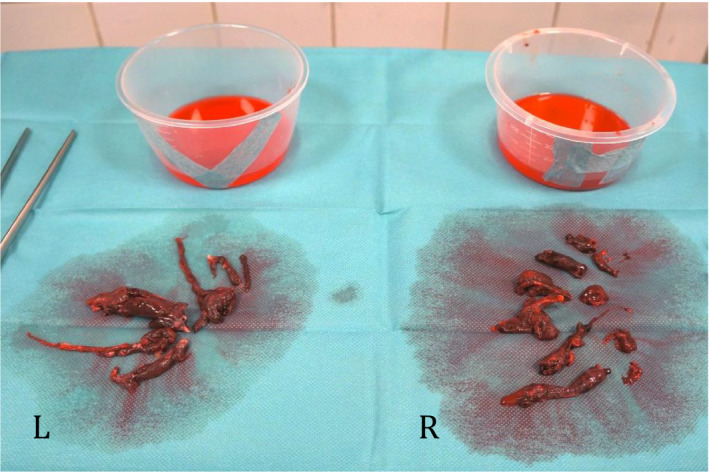
Massive organized thrombus materials were removed from the left (L) and right (R) pulmonary arteries by surgical pulmonary embolectomy

**Figure 5:**
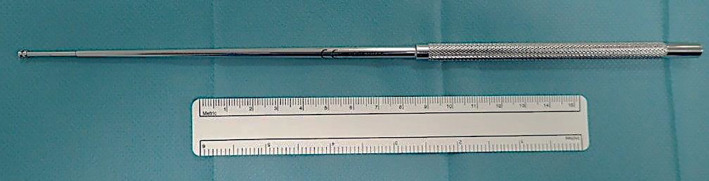
A cardiac suction (Jamieson Dissection Cannulas, Fehling, Acworth, GA 30 101) routinely used for removal of chronic thrombus material in chronic thromboembolic pulmonary hypertension (CTEPH), was successfully used for removal of thrombus in the current case

Briefly, a median sternotomy was performed followed by heparinization and cardiopulmonary bypass with bicaval cannulation. On “beating heart”, a longitudinal arteriotomy was made in the right pulmonary artery, between the aorta and vena cava superior. Through the incision, large amounts of organized thrombus material were successfully extracted and aspirated by forceps and suction (Figure 4). A cardiac suction (Jamieson Dissection Cannulas, Fehling, Acworth, GA 30 101) routinely used for removal of chronic thrombus material in chronic thromboembolic pulmonary hypertension was successfully used for removal of the most peripheral clots (Figure 5). The arteriotomy was closed with prolene 5‐0. A longitudinal incision was now made in the main stem of the pulmonary artery and the procedure repeated as described above.

Postoperatively, the patient was transferred to the intensive care unit (ICU) for recovery. His hemodynamics improved rapidly, and vasopressor and inotropic therapy was de‐escalated during the following two days. Postoperative echocardiography demonstrated full RV recovery with normalization of right/left ventricle ratio. Long‐term oral anticoagulation treatment with vitamin K antagonist was established. The patient was discharged 14 days later from the cardiothoracic ward to neurorehabilitation care with minor cognitive deficits and functional limitations.

## 5 DISCUSSION

Acute pulmonary embolism (PE) is a serious presentation of venous thromboembolism.^1^ Acute obstruction of the pulmonary circulation may increase right ventricular (RV) afterload and cause acute RV failure and shock or sudden cardiac death. Early diagnosis and optimal management are essential for survival with good clinical outcome.

Computed tomography (CT) pulmonary angiography remains the first‐choice diagnostic imaging modality^2^ in hemodynamically stable patients with clinically suspected PE. CT has high sensitivity and specificity for diagnosing PE^3^ and adds valuable prognostic information regarding RV dysfunction, which is a major predictor of mortality in PE.^4^ Pulmonary angiography is considered “gold standard” for diagnosis of PE^5^; however, it is rarely performed due to the availability and high diagnostic performance of CT. Patients presenting with acute high‐risk PE and shock may also be diagnosed with CT; however, in selected patients, pulmonary angiography may be considered. That may include unstable patients admitted directly to the catheterization laboratory at tertiary units, where pulmonary angiography is readily available and transfer to CT may delay diagnosis and increase overall risk. Patients with severe hemodynamic instability and suspected PE with possible need of ECMO support may also be diagnosed by pulmonary angiography in the catheterization laboratory, where mechanical support can be established immediately as a bridge to reperfusion treatment.

Patients diagnosed with acute pulmonary embolism and presenting with sustained hypotension and cardiogenic shock are considered high risk.^5^ Overall, in‐hospital mortality rate of 65% has been found in the subgroup of patients presenting with circulatory collapse and cardiac arrest.^6^ Therapeutic strategy should be decided individually. If necessary, the decision should be made following MDT decision, but a streamlined setup for acute MDT decision‐making is key to shorten decision delay in hemodynamically unstable patients.^7^ Reperfusion treatment should aim to reverse pulmonary vascular obstruction and reduce RV afterload and increase cardiac output. Thrombolytic therapy reduces overall mortality and PE recurrence in patients with acute high‐risk PE^8^ and is the recommended first‐line treatment if no contraindications exist. In case of absolute or relative contraindications to thrombolytic therapy, emergent mechanical removal of thrombus by surgical pulmonary embolectomy should be considered. Surgical embolectomy promptly removes emboli and reduce RV afterload that favors recovery. In a previous study, patients with high‐risk PE treated with surgical pulmonary embolectomy had low perioperative mortality of 5.3% with a high 10 years survival rate of 83.5%.^9^ Rescue surgical pulmonary embolectomy should also be considered in nonresponders with persisting hemodynamic instability following thrombolysis.

Future treatment alternatives may include percutaneous catheter‐directed treatment with thrombus aspiration or in‐situ reduced‐dose thrombolysis, which has shown favorable results in intermediate‐high‐risk PE, but so far results remain to be confirmed by randomized trials in larger patient populations with high‐risk PE.

## 6 FOLLOW‐UP

At present, the patient has survived more than one‐year.

## 7 CONCLUSION

Acute PE can be rapidly diagnosed by pulmonal angiography in the critically ill patient and acute MDT decision‐making followed by emergency surgical embolectomy may favor quick recovery and positive clinical outcome in selected patients with massive PE presenting with cardiogenic shock or OHCA.

## CONFLICT OF INTEREST

None declared.

## AUTHOR CONTRIBUTIONS

ME: performed visitation and first physical examination, including acute echocardiography, participated in MDT conference, and wrote first draft of the original manuscript. AA: participated in MDT conference and editing of the case report. HE: participated in MDT conference and editing of the case report. KEK: performed acute surgical pulmonary embolectomy and participated in MDT conference and writing of case report. TT: performed acute coronary and pulmonary angiography and participated in MDT conference and writing of the case report.

## ETHICAL APPROVAL STATEMENT

Patient consent has been obtained.
